# Phylogenetically informative mutations in genes implicated in antibiotic resistance in *Mycobacterium tuberculosis* complex

**DOI:** 10.1186/s13073-020-00726-5

**Published:** 2020-03-06

**Authors:** Matthias Merker, Thomas A. Kohl, Ivan Barilar, Sönke Andres, Philip W. Fowler, Erja Chryssanthou, Kristian Ängeby, Pontus Jureen, Danesh Moradigaravand, Julian Parkhill, Sharon J. Peacock, Thomas Schön, Florian P. Maurer, Timothy Walker, Claudio Köser, Stefan Niemann

**Affiliations:** 1 German Center for Infection Research (DZIF), Partner site Hamburg-Lübeck-Borstel-Riems, Borstel, Germany; 2 Molecular and Experimental Mycobacteriology, Research Center Borstel, Parkallee 1, 23845 Borstel, Germany; 3 National and WHO Supranational Reference Center for Mycobacteria, Research Center Borstel, Borstel, Germany; 4 Nuffield Department of Medicine, John Radcliffe Hospital, University of Oxford, Oxford, UK; 5 Department of Clinical Microbiology, Karolinska University Hospital, Solna, Stockholm, Sweden; 6 Department of Laboratory Medicine, Karolinska Institute, Stockholm, Sweden; 7 Department of Clinical Science and Education, Emergency Medicine, Stockholm South General Hospital, Karolinska Institute, Stockholm, Sweden; 8 Public Health Agency of Sweden, Solna, Sweden; 9 Center for Computational Biology, Institute of Cancer and Genomic Sciences, University of Birmingham, Birmingham, UK; 10 Department of Veterinary Medicine, University of Cambridge, Cambridge, UK; 11 Department of Medicine, University of Cambridge, Cambridge, UK; 12 Department of Infectious Diseases and Clinical Microbiology, Kalmar County Hospital, Kalmar, Sweden; 13 Department of Clinical and Experimental Medicine, Division of Medical Microbiology, Linköping University, Linköping, Sweden; 14 Institute of Medical Microbiology, Virology and Hospital Hygiene, University Medical Center Hamburg-Eppendorf, Hamburg, Germany; 15 Department of Genetics, University of Cambridge, Cambridge, UK

**Keywords:** *Mycobacterium tuberculosis*, Drug resistance, Benign mutations, Intrinsic resistance

## Abstract

**Background:**

A comprehensive understanding of the pre-existing genetic variation in genes associated with antibiotic resistance in the *Mycobacterium tuberculosis* complex (MTBC) is needed to accurately interpret whole-genome sequencing data for genotypic drug susceptibility testing (DST).

**Methods:**

We investigated mutations in 92 genes implicated in resistance to 21 anti-tuberculosis drugs using the genomes of 405 phylogenetically diverse MTBC strains. The role of phylogenetically informative mutations was assessed by routine phenotypic DST data for the first-line drugs isoniazid, rifampicin, ethambutol, and pyrazinamide from a separate collection of over 7000 clinical strains. Selected mutations/strains were further investigated by minimum inhibitory concentration (MIC) testing.

**Results:**

Out of 547 phylogenetically informative mutations identified, 138 were classified as not correlating with resistance to first-line drugs. MIC testing did not reveal a discernible impact of a *Rv1979c* deletion shared by *M. africanum* lineage 5 strains on resistance to clofazimine. Finally, we found molecular evidence that some MTBC subgroups may be hyper-susceptible to bedaquiline and clofazimine by different loss-of-function mutations affecting a drug efflux pump subunit (MmpL5).

**Conclusions:**

Our findings underline that the genetic diversity in MTBC has to be studied more systematically to inform the design of clinical trials and to define sound epidemiologic cut-off values (ECOFFs) for new and repurposed anti-tuberculosis drugs. In that regard, our comprehensive variant catalogue provides a solid basis for the interpretation of mutations in genotypic as well as in phenotypic DST assays.

## Background

Drug-resistant *Mycobacterium tuberculosis* complex (MTBC) strains are estimated to account for one third of all deaths due to antimicrobial resistance globally [1]. Owing to the inherently slow growth rate of MTBC, the only realistic way to diagnose the majority of drug-resistant cases is to use rapid genotypic drug-susceptibility testing (gDST), which ranges from targeted assays to whole-genome sequencing (WGS) [2]. In fact, it is becoming increasingly clear that gDST assays are better suited than phenotypic DST (pDST) to rule-in resistance caused by known mechanisms that only confer modest minimum inhibitory concentration (MIC) increases, such as for ethambutol (EMB) [3–5].

The accuracy of gDST depends on the ability to distinguish valid markers for resistance (i.e. mutations that directly confer resistance or, alternatively, play a compensatory role in resistance) from neutral mutations that do not alter the susceptibility to an antibiotic [6, 7]. In this context, one of the major confounders is the pre-existing variation in genes associated with resistance, which comprises neutral mutations and, more rarely, changes that confer intrinsic/natural resistance (i.e. resistance that arose by chance/genetic drift prior to the clinical use of a drug or a related agent with a shared resistance mechanism) [8]. Because MTBC displays a strictly clonal population structure without any lateral gene transfer, these mutations are typically phylogenetically informative and unique (i.e. they are markers for a particular subgroup of the global MTBC diversity). Consequently, they form a barcode that is exploited by some targeted gDST assays to provide an epidemiological typing result at no additional cost, albeit at a limited resolution compared with WGS [9–11]. By contrast, homoplastic mutations have arisen multiple times independently in the MTBC phylogeny and are, consequently, not markers for a single subgroup. If this diversity is not considered at the design stage of a gDST assay, they can result in systematic false-resistant results. Indeed, the World Health Organization (WHO) has just revised the reporting language for line probe assays to reflect this possibility (e.g. *gyrA* A90G causes false-resistance reports for fluoroquinolones with the Hain GenoType MTBDR*sl* assay) [12, 13].

The purpose of this study was, therefore, to catalogue phylogenetically informative mutations in 92 genes implicated in the resistance to a total of 21 antibiotics and, where possible, to identify neutral mutations amongst these changes by taking evolutionary information and pDST data into consideration. Moreover, we searched for evidence of previously unknown intrinsic resistance.

## Methods

### Strain collection

We analysed 405 phylogenetically diverse MTBC genomes, of which 214 were drawn from Comas et al. who studied the evolutionary history of MTBC using isolates from 46 countries [14]. This collection was supplemented with mostly pan-susceptible strains from the Research Center Borstel (*n* = 69) [10] and the Karolinska University Hospital in Sweden (*n* = 122) [4].

### WGS

WGS at the Research Center Borstel was performed with Illumina Technology (MiSeq, NextSeq 500, HiSeq 2500) using Nextera XT library preparation kits as instructed by the manufacturer (Illumina, San Diego, CA, USA). Fastq files (raw sequencing data) for all strains analysed in this study are available from the European Nucleotide Archive, and details can be found within Additional file 1: Table S1. All genomes were analysed with the MTBseq pipeline [15]. First, reads were mapped to the *M. tuberculosis* H37Rv genome (GenBank ID: NC_000962.3) with BWA [16]. Alignments were then refined with the GATK [17] and Samtools [18] toolkits for base quality recalibration and alignment corrections for possible PCR and InDel artefact. Variants (SNPs and InDels) were called if the following criteria were met: a minimum coverage of four reads in both forward and reverse orientation, four reads calling the allele with at least a phred score of 20, and an allele frequency of 75%. Deletions in *Rv1979c* were identified manually as the above algorithms are not optimised to call large InDels.

### Phylogenetic analysis

Regions annotated as repetitive elements (e.g. PPE and PE-PGRS gene families), InDels, multiple consecutive SNPs in a 12-bp window (possible InDel artefacts or rare recombination scars), and 92 genes implicated in antibiotic resistance (Additional file 2: Table S2) were excluded for the phylogenetic reconstruction. In the combined analysis, we considered all genome positions that fulfilled the aforementioned criteria for coverage and variant frequency in 95% of all samples in the datasets as valid and used the concatenated sequence alignment to calculate a maximum likelihood tree with Fast Tree [19], employing a GTR substitution model, 1000 resamples, and Gamma20 likelihood optimisation to account for rate heterogeneity amongst sites. The consensus tree was rooted with the “midpoint root” option in FigTree [20], and nodes were arranged in increasing order. MTBC strains were stratified into lineages and subgroups using the classification schemes by Coll et al. and/or Merker et al. [9, 21].

In the ML tree, we identified internal nodes/branches with very good statistical support (bootstrap values ≥ 0.9). Strains derived from one shared internal branch (i.e. most common recent ancestor) were assigned to groups, and group-specific mutations were extracted considering sequences of 92 genes implicated in antibiotic resistance (Additional file 2: Table S2).

Phylogenetic (branch-specific) mutations were further classified using pDST data (mainly MGIT 960) for the first-line drugs rifampicin (RIF), isoniazid (INH), EMB, and pyrazimanide (PZA) from the CRyPTIC consortium [7]. A mutation was regarded as likely neutral if > 90% of strains that did not harbour known resistance mutations were phenotypically susceptible, provided that pDST results were available for at least 10 strains.

### MIC measurements

MIC values for INH (Sigma-Aldrich, Germany), prothionamide (PTO; Riemser, Germany), bedaquiline (BDQ; Janssen, USA), and clofazimine (CFZ; Sigma-Aldrich, Germany) were determined in the BACTEC MGIT 960 system (Becton Dickinson) in conjunction with the EpiCenter TBeXiST software. The following drug concentrations were tested: 0.0125, 0.025, 0.05, 0.1, and 0.4 μg/ml for INH; 0.3125, 0.625, 1.25, 2.5, and 5 μg/ml for PTO; and 0.0625, 0.125, 0.25, 0.5, 1, and 2 μg/ml for BDQ and CFZ. A strain was interpreted as resistant to a drug at a particular concentration if the drug-containing tube reached ≥ 100 growth units before the drug-free control tube with the 1:100 diluted suspension of the strain reached 400 growth units.

## Results

### MTBC strain collection and phylogeny

Our collection (*n* = 405) featured 296 evolutionary “modern”, i.e. TbD1 region deleted [22], *M. tuberculosis* strains (i.e. lineages 2–4), and 109 evolutionary “ancestral” (TbD1 region intact) MTBC lineages, ranging from lineages 1 and 7 (*M. tuberculosis*, *n* = 60) and lineages 5 and 6 (*M. africanum*, *n* = 35) to 14 animal-adapted species (i.e. *M. pinnipedii*, *M. microti*, *M. orygis*, *M. caprae*, *M. bovis*, including one *M. bovis* BCG vaccine strain (Additional file 1: Table S1). Of these isolates, only 40 (9.9%) had a mutation in the RIF resistance determining mutation in *rpoB* and were, consequently, RIF resistant.

**Fig. 1:**
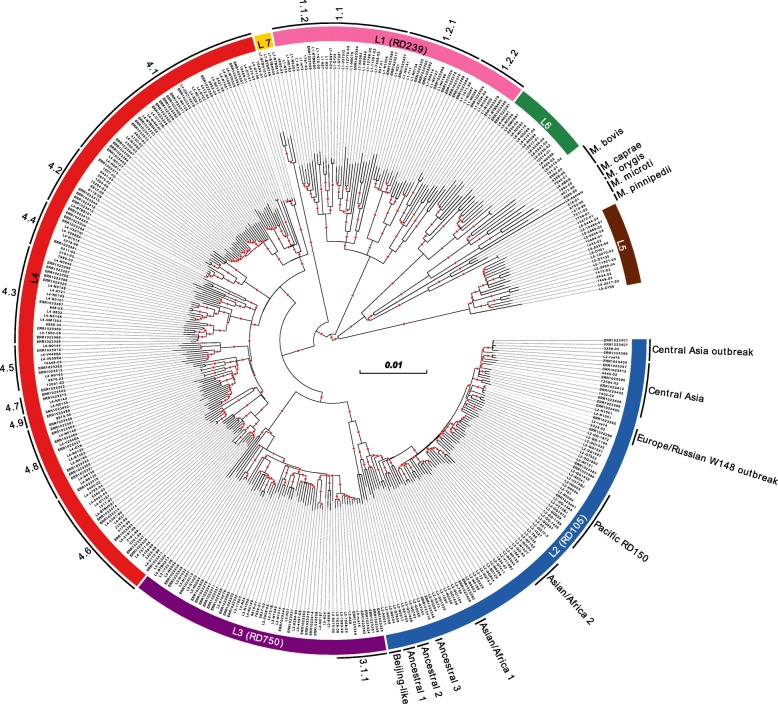
MTBC phylogeny. ML phylogeny based on 42,760 SNPs from 405 genomes using a general time-reversible substitution model and 1000 resamples. Seven major MTBC lineages and animal-adapted species are highlighted. Where warranted, these were differentiated further into subgroups (as shown on the circumference of the figure). Red dots indicate branches (*n* = 334) with a resampling support of > 0.9 and which were investigated for branch-specific mutations in 92 genes implicated in antibiotic resistance

A maximum likelihood (ML) tree with a general time-reversible (GTR) substitution model and 1000 resamples was calculated based on 42,760 single nucleotide polymorphisms (SNPs), excluding mutations in repetitive regions and 92 genes implicated in antibiotic resistance (Additional file 2: Table S2). Next, we identified all internal nodes/branches with a bootstrap support of 0.9 and higher (*n* = 334). All isolates that shared a common node/branch with sufficient bootstrap support were grouped together, and group-specific changes, i.e. SNPs and insertions and deletions (InDels), in the aforementioned 92 genes were identified (Fig. 1).

### Phylogenetically informative and unique mutations: an overview

We found a total of 557 group-specific mutations in the 92 genes that had either been implicated in antibiotic resistance, are confirmed resistance mechanisms, or are involved in compensating for the reduced fitness of resistance mutations in other genes. Out of 557 mutations, we excluded 26 mutations as they were found in groups that only comprised strains with a pairwise distance of up to five SNPs, suggesting recent transmission. However, three H37Rv-specific mutations (4.9 subgroup) and the aforementioned *gyrA* A90G change (4.6.1.2 subgroup) were exempt from this rule given their phylogenetic importance [12]. This list of mutations was further supplemented with three known phylogenetic mutations (i.e. *tlyA* N236K in the 4.6.2 subgroup [23], *cycA* G122S in BCG, and *mmaA3* G98D in a subgroup of BCG [8]) that confer intrinsic antibiotic resistance (Additional file 3: Table S3). These three mutations were not highlighted by our algorithm because they featured only in single strains in our collection. Where possible, we compared the resulting 539 mutations with the typing scheme by Coll et al., the most widely used method to stratify WGS data for MTBC [9]. Of the 89 SNPs that were in common, 82 results were in agreement. Subsequent personal communications with Francesc Coll revealed that the seven discrepancies were due to errors in his study and were eventually resolved (Additional file 3: Table S3).

### Phylogenetically informative and unique mutations: impact on resistance

We then proceeded to identify mutations that were likely neutral (i.e. do not correlate with resistance) using previously published pDST data for RIF, INH, EMB, and PZA for over 7000 isolates [7], yielding 138 neutral mutations (Additional file 3: Table S3).

Notably, we identified five group-specific mutations that had been linked with drug resistance in the literature. This included the *ndh* R268H mutation, which had been proposed as an isoniazid resistance marker, and we classified it as a marker for a subgroup of lineage 1.1.2 [24]. Yet, our analysis of routine pDST data and additional MIC testing demonstrated that *ndh* R268H was likely neutral (Additional file 4: Tables S4). Moreover, we undertook MIC testing to investigate the roles of the *ethA* M1R, S266R, and G413D mutations, which had been previously associated with PTO and ethionamide (ETO) resistance (Additional file 4: Table S4) [25–27]. Of these mutations, only isolates with the *ethA* M1R mutation tested resistant to PTO, which was expected as this mutation should abolish the start codon of *ethA*. This particular mutation was shared by four isolates that formed a subgroup within lineage 4.2.2 (TUR genotype) with a median pairwise genetic distance of 22 SNPs and were at least resistant to INH. Consequently, it was unclear whether this mutation arose in response to the exposure to PTO/ETO or whether this represented an example of intrinsic resistance.

In addition, we measured the CFZ MICs for the *Rv1979c* V52G mutation, which yielded MICs in the susceptible range (Additional file 4: Table S4). This result, therefore, supported a recent study that found that this alteration, which was shared by a group of two Beijing isolates, probably does not confer CFZ resistance [28].

Our analysis supported the hypothesis that the entire MTBC branch that comprises both *M. africanum* lineages and the animal-adapted strains (*M. africanum*/animal branch) likely has intrinsically elevated MICs to cycloserine (DCS), owing to a 1-bp frameshift deletion in *ald*, which encodes alanine dehydrogenase (Additional file 3: Table S3) [5, 29]. In addition, we expect the DCS MIC to be raised further in *M. microti* and *M. pinnipedii* given that both species harbour a frameshift in *cycA*, which should impede DCS uptake, as is the case in all BCG variants due to a G122S mutation in the same gene [5, 29, 30].

In contrast to these cases, this study also raised the prospect that some subgroups may be more susceptible to particular antibiotics due to their specific genetic background. For example, we observed different loss-of-function (LOF) mutations in *mmpL5* in two genetic backgrounds (in subgroups of lineages 1.1.1.1 and 4.6) that should render them hyper-susceptible to bedaquiline (BDQ) and CFZ [31]. Moreover, we observed LOF mutations in *eis* and its transcriptional activator *whiB7*, which might make the respective genotypes more susceptible to kanamycin (KAN) [4, 32]. Finally, we confirmed that most lineage 2 strains share a frameshift in the *tap* efflux pump, which means that mutations that result in the overexpression of *whiB7* cannot confer streptomycin (STR) resistance in these strains [33].

### Convergent evolution: impact on resistance

We observed 27 changes that had evolved independently in multiple genetic backgrounds and, consequently, were not markers for only one phylogenetic group (Additional file 5: Table S5), which is typically a sign of positive selection [34]. Indeed, several classical resistance mutations featured in this category, which were likely selected in response to antibiotic treatment (e.g. *rpoB* S450L and *rpsL* K43R). Another well-understood mutation affected codon 220 of *pykA*. A glutamic acid to aspartic acid change, which results in the inability to grow on glycerol as the sole carbon source, occurred at the base of the *M. africanum*/animal branch [35]. It has already been reported that some strains within this group (i.e. *M. suricattae* and all BCG variants) independently regained the ability to grow on glycerol by reverting back to glutamic acid [29]. Here, we found that this occurred on two more occasions, i.e. in one lineage 6 strain (L6-N0060) and in our variant of *M. bovis* ATCC 19210 (9564-00) [36].

As previously reported, *Rv1979c* was deleted independently in all *M. africanum* lineage 5 strains (del *Rv1978*-*Rv1979c* [37]), in our two *M. pinnipedii* strains (del *Rv1964*-*Rv1979c* [38]), and also in more recently derived BCG variants (*Rv1964-Rv1988*, i.e. RD2) [39, 40]. Given that mutations in *Rv1979c* are implicated in CFZ resistance, this raised the possibility that intrinsic resistance to CFZ might have arisen on at least three occasions in MTBC [5, 28, 41]. However, testing of five *M. africanum* lineage 5 strains did not reveal a discernible increase of the CFZ MIC (Additional file 4: Table S4).

There was at least one example where convergent evolution was misleading. The ancestral nucleotide at position -32 upstream of *ald* is adenine whereas glycine was acquired independently by *M. africanum* lineage 5 and a group of lineage 4 strains (i.e. 4.5, 4.6, 4.7, 4.8, and 4.9). Yet, because *ald* is inactive in lineage 5, as mentioned above, this mutation cannot have the same effect in both genetic backgrounds.

## Discussion

Compared with most bacterial pathogens, MTBC is monomorphic [42]. Nevertheless, it has been known for more than 60 years that this limited diversity can result in intrinsic resistance [43]. Indeed, if resistance to an antibiotic can arise by LOF mutations that do not have major adverse consequences for bacterial fitness, it is not a question of whether intrinsic resistance exists but, rather, how widespread this phenotype is. This, in turn, is a function of how deeply rooted this phenotype is in the phylogenetic tree of MTBC and how well these strains have subsequently transmitted. For example, the *pncA* H57D mutation, which is estimated to have evolved approximately 900 years ago [44], is shared by the vast majority of *M. bovis* strains and consequently renders them intrinsically resistant to PZA [45]. Yet, owing to the control policies introduced in well-resourced countries over the past century, *M. bovis* is responsible for fewer than 3% of human tuberculosis (TB) cases globally [46].

The remaining experimentally confirmed cases of intrinsic resistance have arisen more recently in MTBC and, therefore, are less frequent. These include the intrinsic capreomycin resistance of some lineage 4.6.2 strains (Cameroon genotype) due to the *tlyA* N236K mutation [23], the high-level DCS resistance shared by all BCG variants [29], and the intrinsic resistance to INH and ETH/PRO of BCG variants derived after 1926 as a result of *mmaA3* G98D [39]. Finally, *M. canettii*, which is not strictly speaking part of MTBC, is intrinsically resistant to PZA and pretomanid [45, 47, 48].

Yet, the molecular evidence from this study underscores that even more deeply rooted and thus older instances of intrinsic resistance have either not been studied sufficiently or may have been missed completely. The possibility that the entire *M. africanum*/animal branch likely has intrinsically elevated MICs to DCS is particularly concerning in light of the severe toxicity of DCS and terizidone [5, 29]. More MIC data are urgently needed to inform pharmacokinetic/pharmacodynamic (PK/PD) modelling to set a clinical breakpoint for DCS and to assess whether this increase is clinically relevant [49].

Conversely, genetic diversity may also confer hyper-susceptibility, which has not been studied systematically in the TB field as MICs are typically truncated at the lower end, i.e. sufficiently low concentrations are not typically tested to define the lower end of “susceptible” MIC distributions [5].

MIC testing of *M. africanum* lineage 5 strains did not confirm the role of the deletion of *Rv1979c* in CFZ resistance, as previously hypothesised [5]. This apparent contradiction might be explained if only specific gain-of-function mutations as opposed to LOF mutations in this gene, which includes a possible permease, confer resistance [28, 41]. Knockout and complementation experiments are currently ongoing to investigate this question further.

Finally, epistatic interactions may affect the way in which mutations are interpreted, as illustrated by the effect of *whiB7* promoter-up mutations, which confer cross-resistance to KAN and STR [32]. However, this is only the case in genetic backgrounds in which both *eis* and *tap* are functional, which is not always the case (e.g. in almost the entire lineage 2) [4, 33].

The number of open questions raised by our study is symptomatic of the lack of rigour used to define breakpoints to anti-TB drugs [3, 5, 50, 51]. The recent endorsement of an MIC reference method by the European Committee for Antimicrobial Susceptibility Testing (EUCAST) and associated guidelines to calibrate other methods, such as the widely used MGIT 960 system, are designed to address these shortcomings [52]. Indeed, these guidelines stipulate that representatives of lineages 1–7 must be tested to define sound epidemiologic cut-off values (ECOFFs). It would be in the interest of pharmaceutical companies to follow the EUCAST guidelines as early as possible during drug development to identify agents that may not be equally effective against major MTBC genotypes [53]. These antibiotics could either be abandoned or their development adjusted to gather evidence that genotypes with intrinsically elevated MICs are treatable at either standard or increased dosing (e.g. using nonclinical models or by choosing clinical trial sites in countries where these genotypes are sufficiently frequent to provide enough statistical power to study these questions comprehensively [54–56]).

## Conclusion

We provide a comprehensive catalogue of phylogenetically informative mutations in genes implicated in drug resistance in MTBC. Our analysis underlines that despite being monomorphic, the genetic diversity in MTBC has to be studied systematically to inform the interpretation of gDST results for existing drugs as well as the development of urgently needed novel agents.

## Supplementary information

**Additional file 1: Table S1.** Metadata for 405 MTBC isolates/datasets used in this study.

**Additional file 2: Table S2.** 92 genes (coding and upstream regions) implicated in antibiotic resistance.

**Additional file 3: Table S3.** Catalogue of 547 phylogenetically informative mutations in 92 genes implicated in drug resistance to 21 anti-TB drugs.

**Additional file 4: Table S4.** MIC results for isolates with *ndh* R268H, e*thA* M1R, e*thA* S266R, e*thA* G413D, or *Rv1979c* V52G mutations, or a *Rv1978*-*Rv1979c* deletion.

**Additional file 5: Table S5.** Overview of homoplasic mutations.

## Data Availability

Fastq files (raw sequencing data) for all strains analysed in this study are available from the European Nucleotide Archive, and details can be found within Additional file 1: Table S1.
